# Technological writing interventions in mental health occupational therapy: considerations for ethical and effective practice

**DOI:** 10.3389/fpsyt.2025.1663697

**Published:** 2025-11-18

**Authors:** Kristine Haertl

**Affiliations:** Department of Occupational Therapy, St. Catherine University, St. Paul, MN, United States

**Keywords:** mental health, therapeutic writing, ethics, creative writing, personal writing

## Abstract

The application of creative and therapeutic writing interventions within mental health occupational therapy remains underexplored in the current literature. Occupational therapists frequently adapt approaches from related disciplines, such as psychology, to develop interventions for individuals with mental health conditions. With the increasing integration of digital tools, including online journals, blogs, and artificial intelligence (AI), there is a growing opportunity to enhance occupational therapy practices through creative and expressive writing. This paper provides a concise review of relevant literature, examines current and emerging uses of creative and therapeutic writing in occupational therapy, and explores key practical and ethical considerations for both therapists and clients.

## Introduction

The integration of technology-based writing interventions in mental health occupational therapy has undergone significant evolution. Across cultures and throughout history, written symbols and forms have served as powerful tools for expression and reflection. Writing, in its various forms, has long been recognized for its therapeutic benefits (1). With the advent of digital technology, traditional practices such as journaling, personal and creative writing, and therapeutic writing have expanded to include blogs, online platforms, social media, and even artificial intelligence. Occupational therapists have traditionally drawn on research and practices from other disciplines such as psychology to inform their use of therapeutic writing interventions. This paper presents practical examples of how mental health occupational therapists can incorporate contemporary, technology-supported writing practices to enrich therapeutic outcomes.

## Examples of evidence and occupational therapy applications

The therapeutic use of writing has been significantly shaped by psychologists such as Ira Progoff, who advocated for journal writing as a tool for self-expression and personal development (2, 3), and James Pennebaker, whose research demonstrated that brief writing sessions (15–20 minutes) focused on emotionally significant experiences can lead to measurable improvements in physical and psychological health (4–7). Studies have also highlighted the healing potential of writing for individuals with mental illness (4, 8), the value of creative writing in processing emotionally charged events (e.g., 4, 5, 8, 9), and the role of therapeutic writing in enhancing client autonomy and strengthening the therapeutic alliance (9).

While much of the research on the health benefits of writing originates from the psychological and social sciences, its application within the field of occupational therapy has received comparatively little attention. The following are illustrative examples of the use of writing in occupational therapy. Cooper (10, 11) investigated the use of a six-session manualized writing program, *Using Writing as Therapy* (UWAT), in mental health settings. Consistent with findings from other disciplines, her work suggests that therapeutic writing can yield personal health benefits when integrated into clinical practice. Haertl (8, 12) examined the role of personal writing in self-development and its healing potential for individuals with mental health conditions. Her findings highlighted the individualized, spiritually transcendent, and dynamic nature of writing; its capacity to support perspective-taking; its therapeutic properties; and its significance in fostering personal growth.

Building on this work, Haertl (1) proposed practical strategies for incorporating therapeutic writing into evaluation and intervention within occupational therapy. She emphasized that writing interventions should be aligned with the theoretical models guiding practice. For example, free writing and flow writing are well-suited to a psychodynamic frame of reference, while structured writing tasks may align more closely with cognitive behavioral models. Similarly, writing could be situated in the Model of Human Occupation through occupational reflections, or the mapping of one’s daily time use patterns. Consequently, the effective use of writing in occupational therapy requires not only familiarity with specific writing methods, but also a clear understanding of the theoretical frameworks that inform their application.

## Integrating technological and virtual applications to therapeutic writing

The integration of technology in therapeutic writing exists along a continuum, from low-tech approaches, such as specialized writing utensils and craft-based methods, to emerging high-tech tools such as artificial intelligence (AI), which is in its early stages within the mental health field. In occupational therapy, the dynamic interaction between the client, their environment, and the occupation can serve as a valuable framework for guiding the use of technological tools in writing based interventions. This integration can support the development of individualized evaluations, intervention plans, and may inform service delivery strategies, such as designing group sessions for specific populations in particular settings. While technology and AI hold promise throughout the occupational therapy process in use of therapeutic writing, their implementation must ensure that therapists receive appropriate training and adhere to ethical standards.

The decision to implement therapeutic writing interventions in-person or virtually depends on several factors, including client needs and preferences, resource availability, geographic location, and scheduling logistics. While some clients may benefit from the structure and connection of in-person sessions, others, particularly those with agoraphobia, mobility limitations, or transportation challenges, may find virtual formats preferable and more accessible.

When planning a therapeutic writing intervention online, several key considerations should guide decision-making:

- Format and Delivery: Will the sessions be delivered individually or in a group setting? Will the format be synchronous (real-time), asynchronous (self-paced), or a hybrid model?
- Scheduling Logistics: How will sessions be scheduled, particularly in group formats with participants across different time zones?
- Therapeutic Containment and Privacy: What measures will ensure confidentiality and emotional safety? For example, many online journal platforms offer privacy settings such as “private,” “friends only,” or “public” to control content visibility.
- Therapist Qualifications: What is the therapist’s training in therapeutic writing, and how prepared are they to manage sensitive topics that may surface during sessions?
- Ownership of Written Content: What happens to the writing after sessions conclude? Does it remain with the client, the therapist, the group, or is it shared in another agreed-upon format?
- Therapeutic Goals: What are the specific goals for both the group and individual participants? How will writing activities support these outcomes?

## The role of technology in occupational therapy writing interventions

Research on the use of technology-based, online therapeutic writing is still in its early stages. Studies indicate that this approach may support psychological well-being and stress reduction, particularly among socially inhibited individuals (13). Additionally, online therapeutic writing has shown potential in helping clients process grief (14) and, consistent with previous findings, foster personal transformation and growth (15).

Haertl (1) described writing techniques within the psychodynamic/object relations frame of references to include free writing, flow writing, expressive writing, stream of consciousness, and dream exploration, among other techniques. Within expressive writing, at times the topic may flow freely from the client, at other times writing prompts may be used to facilitate the writing process. When using free writing, it is important to consider whether the writing will be open ended or time limited and how it will facilitate client insight and goal attainment.

In these techniques, clients are encouraged to freely express their thoughts and emotions without self-judgment or concern for grammar or structure. The therapist collaborates with the client to determine what content, if any, will be shared with the therapist or others, and how personal insights might be drawn from the written expression.

When considering therapeutic writing methods, such as Pennebaker’s prescriptive writing for processing emotional events, the following guidelines may be helpful:

- Find a locale where the client won’t be disturbed (online venues can be helpful for this)
- Decide whether the topic will be similar or different each time of writing; find topics that have brought up emotions or have caused negative health effects- work to come to understand these events and develop a strategy for healing
- Determine with whom (if anyone) the writing is shared
- Consider whether the writing will be saved or destroyed

In addition to expressive and creative forms of writing (e.g. poetry, story making), writing activities may be more in line with a cognitive behavioral focus and utilized structured exercises, lists, clustering and homework (1). In addition, Progoff (2, 3) introduced the use of dialogue to explore the relationship to self and others. Topics of unfinished business such as a conflict with a friend are explored via having the client write from the perspective of all the individuals involved. Consideration of readiness to explore the situation along with the ability of the client to gain emotional release or insight from the dialogue should be considered. Key insights may emerge that could be addressed further in occupational therapy such as coping skills, stress management, interpersonal skills, and the incorporation of occupational activities to address health and wellness.

Another writing-based intervention which may be delivered in person or online includes *Using Writing as Therapy* (10, 11), developed by Cooper, utilizes a six-session format focused on identity exploration and self-esteem. Unlike the free-form writing described above, this format utilizes therapist prompts which may focus on emotional disclosure and distancing in order to gain insights from the writing through reframing and coming to understand events in new ways. The format may be delivered in a group setting and offer participant sharing and feedback. The therapist closes the group with discussion and possible homework prior to the next session. Research suggests this format may lead to improved self-knowledge, cognitive change, and enhanced clarity of identity.

The following describes additional potential applications for technological use of writing-based interventions:

Blogs: Blogs are an online venue, often public in nature, that individuals share about topic(s) of personal interest to them. Typically entries are posted in reverse chronological order and depending on the platform may or may not invite commentary and discussion. Similar to other forms of therapeutic writing, blogs may provide a venue for emotional expression and stress reduction, yet consideration should be given to the intended purpose and level of public disclosure. Murphy and colleagues (16) conducted a scoping review exploring mental health issues through the use of blogs. Within blogs in the public sphere, the authors found themes of safety through moderation, confidentiality, and public/private boundaries. The authors asserted that having some level of moderation (e.g., a facilitator) can be important if there is a public sharing and focus on sensitive topics. The authors also found that while confidentiality can be important if there are shared blogs, often if individuals knew each other’s identities it may further build community. In addition, having a facilitator in shared blog spaces for the purpose of therapeutic writing, can often be of benefit in cases when someone may have lost touch with reality, or may venture to a place where the narrative could be harmful, disillusioned, or countertherapeutic to others. Thus, a therapist should carefully consider the pros and cons of the type of blogging and the use of private *vs*. public or shared spaces.

The same scoping review (16) found that within the private sphere blogging may offer catharsis, opportunity to develop coping skills, and social connectedness if the blogging for instance, occurs with a small group of individuals with similar connections, interests, or experiences. Such blog venues may be self-generated, or facilitated by an experienced therapist.

Digital Journaling Options: There are several digital journaling platforms, often offering options to post and store photos, designate the level of privacy (e.g., private, friends, public), and to sort journal entries via calendar and word tags. Recently, the addition of AI powered digital journal options offer additional features beyond traditional journal-based websites. These AI platforms-provide journal prompts and feedback in order to enhance client insight and perspective. Programs such as MindScape and Mindsera use large language models (LLM) that seek to provide a personalized contextualized journal-based experience catered to the user. Similar to other studies on therapeutic writing, research has suggested the utility of utilizing AI powered journal platforms in promoting self-reflection and insight (17).

As with any form of therapeutic writing, the integration of these tools into mental health occupational therapy should be guided by careful consideration of client needs, therapeutic goals, and platform suitability. Importantly, plans for crisis intervention and connection to appropriate support systems must be in place, particularly for clients experiencing acute mental health challenges or requiring ongoing engagement with a therapist or support network. Examples of use of these tools within occupational therapy include the development of a Wellness Recovery Action Plan (WRAP) that highlights meaningful and healthy occupations that promote health and well-being. Another application would include tracking one’s occupational balance, or developing a plan for occupational engagement and recording progress and reflections related to the plan. Digital platforms may also be used to work on self-expression as AI may help facilitate journal or poetry writing, or facilitate personal reflection. It is important to assure that AI helps facilitate autonomy and goal attainment rather than dependence.

Chat Bots: Chatbots are computer-based programs that simulate human interaction. They have been shown to help facilitate improved mental health (18) and may be used in a variety of therapeutic applications including personal writing. Similar to other areas of therapeutic writing, the therapist should be educated on platforms available and consider the client-occupation fit with the goals of the client.

Additional Tools: There are several additional online means to use therapeutic writing in practice. AI generated tools, gamified writing (having gaming elements within the writing process), collaborative writing portals, and use of telehealth in coaching the writing process are all methods of delivery that therapists may use. Similar to that which is discussed above, the client-occupation fit along with therapist training and resources available are all considerations in the planning of therapy.

## Implications for occupational therapy

This article has provided an introduction to the literature and potential use of writing interventions for occupational therapy. The use of technological based writing interventions has potential for growth and future research, the following are considerations for the field of occupational therapy.

Practice Recommendations.

1. Occupational therapists should ground the use of therapeutic writing to practice-based models as discussed above.
2. Individualize writing based interventions based on the client’s interests, values and goal areas.
3. Integrate technology thoughtfully and carefully consider confidentiality (e.g., if the writing will be shared or not).
4. Facilitate reflection on what is learned from the writing.
5. Pursue training in narrative and therapeutic writing, digital platforms and the ethical use of AI.

There is need for future research in technological based writing evaluation and intervention. Areas of exploration include the interdisciplinary use of writing interventions, means to incorporate writing into occupation-based practice, and further exploration of the ethical use of AI tools.

## Conclusion

As the landscape of mental health occupational therapy continues to evolve, creative and therapeutic writing, when enhanced by digital technology offers a underutilized avenue for client engagement, self-expression, and growth. Grounded in evidence, writing-based interventions have demonstrated value in improving emotional well-being, fostering insight, supporting identity development, and enhancing therapeutic outcomes.

This paper has outlined both traditional and emerging approaches to therapeutic writing, including expressive and structured techniques, and has explored how digital platforms such as blogs, online journals, and AI-powered tools can expand accessibility and personalization. These tools when thoughtfully integrated into occupational therapy, can provide clients with meaningful opportunities for catharsis, reflection, social connection, and skill development. However, their use also requires careful ethical and clinical consideration, including privacy, content moderation, therapist training, and crisis support.

Ultimately, the success of writing-based interventions depends on a strong client-occupation fit, alignment with therapeutic goals, and the therapist’s ability to create a safe, supportive environment. As technology continues to advance, occupational therapists are uniquely positioned to innovate in this space, drawing on both evidence-based practices and creative application to empower clients in their mental health journeys through the written word.

## Data Availability

The original contributions presented in the study are included in the article/supplementary material. Further inquiries can be directed to the corresponding author.
